# Minimally invasive hybrid surgery combined with endoscopic and thoracoscopic approaches for submucosal tumor originating from thoracic esophagus

**DOI:** 10.1186/s12957-015-0452-6

**Published:** 2015-02-12

**Authors:** Hiroyuki Daiko, Takeo Fujita, Takahiro Ohgara, Nobuyoshi Yamazaki, Satoshi Fujii, Yasuhiro Ohno, Tomonori Yano

**Affiliations:** Division of Esophageal Surgery, National Cancer Center Hospital East, 6-5-1 Kashiwanoha, Kashiwa, Chiba 277-8577 Japan; Division of Clinical Pathology, National Cancer Center Hospital East, 6-5-1 Kashiwanoha, Kashiwa, Chiba 277-8577 Japan; Division of Gastrointestinal Endoscopy, National Cancer Center Hospital East, 6-5-1 Kashiwanoha, Kashiwa, Chiba 277-8577 Japan

**Keywords:** Submucosal tumor, Thoracoscopy, Gastrointestinal stromal tumor, Esophagus, Esophagoscopy

## Abstract

**Background:**

Despite the efficacy of molecular targeted therapy, surgical resection remains the only curative primary treatment for gastrointestinal stromal tumors (GISTs). However, in cases when the tumor originates from the thoracic esophagus, conventional transthoracic approach is highly invasive.

**Methods:**

All procedures were performed with patients in a prone position through a double-lumen endotracheal tube for single-lung ventilation. First, to clarify the resection layer between the tumor and mucosal layer of the esophagus, a sodium hyaluronate solution colored with indigo carmine was injected into the submucosa via the esophagoscopic approach. Second, we thoracoscopically divided the longitudinal muscle of the esophagus and enucleated the tumor through three ports by dissecting along the artificially colored submucosa, thereby minimizing accidentally opening of the esophageal mucosa. Third, we sutured the divided longitudinal muscle layer and removed the tumor from the thoracic cavity.

**Results:**

Four tumors, including one GIST, were successfully resected via this hybrid approach. The mean surgical time was 137.7 min (range, 60–231 min), and the mean blood loss was 21.2 ml (range, 3–65 ml). No perioperative complications occurred, including with accidental opening of the esophageal mucosa.

**Conclusions:**

Our minimally invasive hybrid surgery combined with esophagoscopic and thoracoscopic approaches demonstrated successful resection. This surgery could have advantages both for curing esophageal submucosal tumor and for minimizing surgical invasiveness.

## Background

Even with the recent advances in diagnostic modalities, a correct diagnosis of submucosal tumors (SMTs) originating from the esophagus is still challenging [1-4]. In cases when the tumor is larger than 3 cm in size, surgical resection is recommended for definitive diagnosis and treatment due to a higher risk of malignancy [1]. For the surgical resection of gastrointestinal stromal tumors (GISTs) originating from the thoracic esophagus, thoracotomy with esophagectomy is still the preferred approach, particularly in cases when the tumor is relatively large in size. However, this procedure is usually accompanying large operation scar with reconstruction of the gastric tube. This results in postoperative pain and a relatively higher incidence of accidental opening of the esophageal mucosa [5,6].

With patients in a prone position, thoracoscopic esophagectomy has been reported to be safe and effective, resulting in sufficient surgical outcomes for cancer of the thoracic esophagus [7]. The thoracoscopic approach with patients in a prone position provides an excellent surgical field that makes dissection easier because the physical retraction of organs is not required. Herein, we present the surgical procedures and outcomes of our minimally invasive hybrid approach combined with esophagoscopy and thoracoscopy for enucleating SMTs originating from the thoracic esophagus.

## Methods

### Human rights statement and informed consent

All procedures followed were in accordance with the ethical standards of the responsible committee on human experimentation and with the Helsinki Declaration of 1964 and later versions. Informed consent or substitute for it was obtained from all patients for being included in the study. Patient data were retrospectively evaluated after receiving approval from our institution’s investigational review board with the approval code of 012-0045.

### Surgical position

All procedures were performed with patients under general anesthesia through a double-lumen endotracheal tube for single-lung ventilation. After intubation, each patient was placed in a prone position on a surgical bean bag, and the patient’s right arm was raised cranially. The surgeon and camera surgeon stood on the patient’s right side. The esophagoscopist stood at the patient’s head (right side), while the endoscopy cart with the monitor was positioned on the other side. Written informed consents were obtained from the patients for the publication of this report and any accompanying images.

### Esophagoscopy

**Figure 1:**
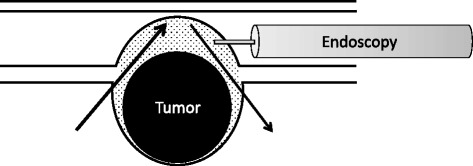
**Schematic representation of esophagoscopic approach and injection of blue-colored sodium hyaluronate solution.** Sodium hyaluronate solution colored blue by indigo carmine was injected into the submucosal layer to define the incision layer and create a space between the tumor and submucosal layer.

Esophagoscopy and thoracoscopy were initiated simultaneously. A single-channel videoscope (Olympus GIF-Q260, Tokyo, Japan) was used for esophagoscopy. A sodium hyaluronate solution (MucoUp™; Seikagaku Corporation, Tokyo, Japan) colored blue by indigo carmine was injected into the submucosal layer to highlight the incision layer and was used to define the space between the tumor and submucosal layer (Figure 1).

### Thoracoscopy

**Figure 2:**
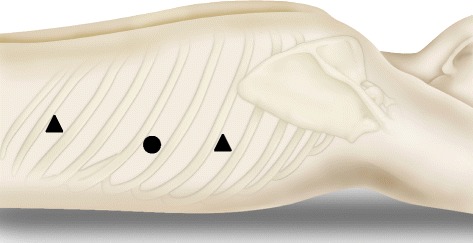
**Schematic representation of the thoracoscopic three-port approach.** Port I is a 12-mm blunt port at the seventh intercostal space (ICS) on the middle axillary line for the left-handed procedure. Port II is a 5-mm port at the fifth ICS on the middle axillary line for the right-handed procedure. Port III is a 5-mm port at the ninth ICS on the posterior axillary line for the insertion of a 5-mm-diameter flexible endoscope.

Thoracoscopy was performed by the surgeon and camera surgeon through three observation ports. Port I was a 12-mm blunt port at the seventh intercostal space (ICS) on the middle axillary line for the left-handed procedure. Port II was a 5-mm port at the fifth ICS on the middle axillary line for the right-handed procedure. Port III was a 5-mm port at the ninth ICS on the posterior axillary line where a 5-mm-diameter flexible endoscope was inserted (Figure 2). Carbon dioxide was usually insufflated at a pressure of 8–10 mmHg to expand the mediastinum.

Thoracoscopy through a three-port approach was sufficient to obtain optical dissection of the submucosal layer, which was artificially colored blue with MucoUp™ (Figure 1).

**Figure 3:**
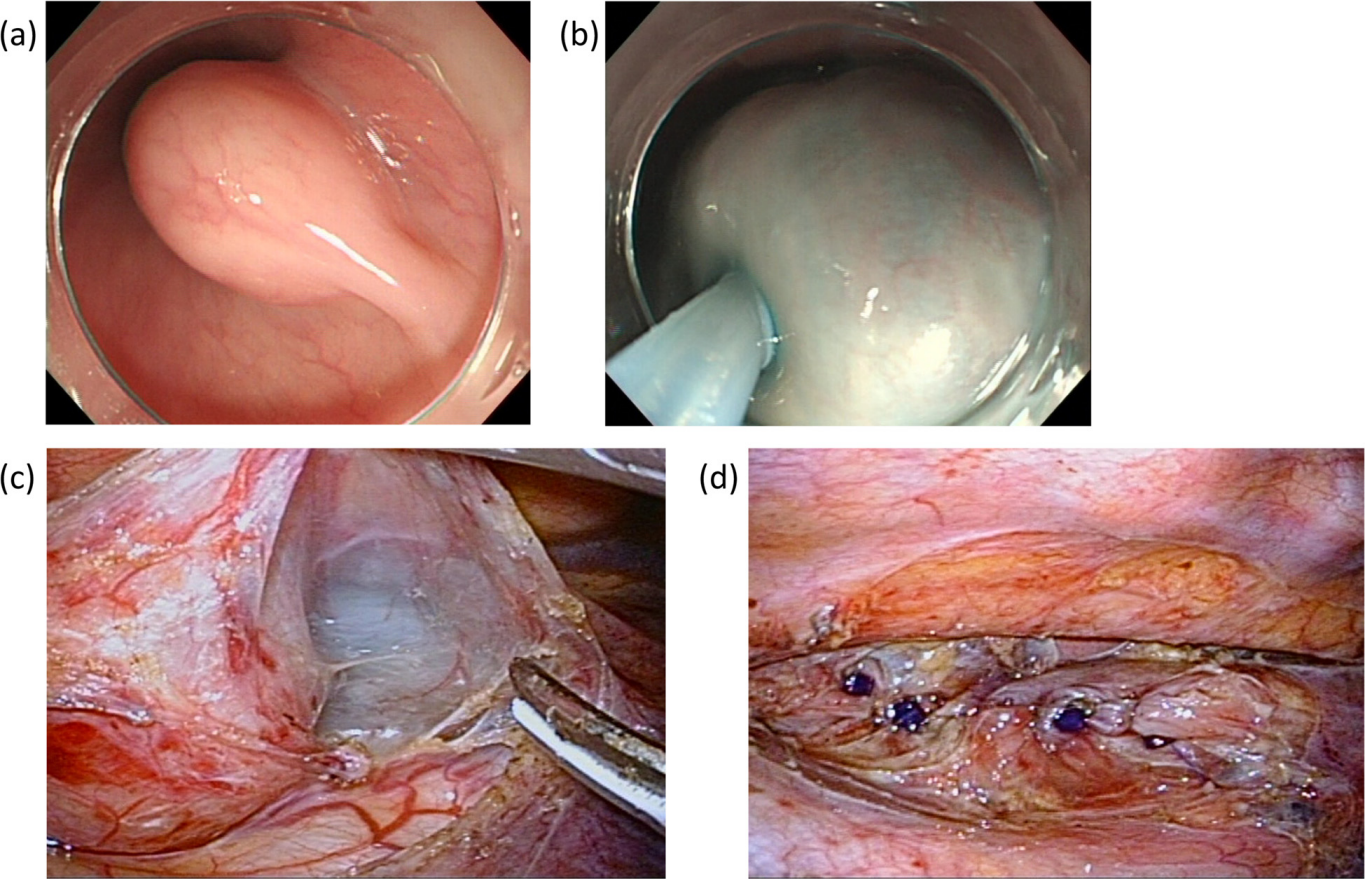
**Representative images of endoscopic and thoracoscopic procedures. (a)** Endoscopic finding of the submucosal tumor before injection of the blue-colored sodium hyaluronate solution. **(b)** Endoscopic finding after the injection of the blue-colored sodium hyaluronate solution. **(c)** Surgical finding after the dissection of the longitudinal muscle layer of the esophagus. **(d)** Surgical finding after the suture of the longitudinal muscle layer of the esophagus.

Thoracoscopy consists of four steps. First, the mediastinal pleura was incised longitudinally along with the ventral border of the azygos vein; this procedure enabled adequate exposure of the tumor. The esophagus was then mobilized from the surrounding tissue to allow a distance of 2 cm from the esophageal tumor to acquire optimal space for the subsequent procedure. Second, the outer longitudinal muscle layer over the tumor was incised and dissected down to the tumor level to preserve the inner circular muscle layer. Third, the internal circular muscle layer was divided, and the thickened submucosal layer marked by indigo carmine was identified. Careful enucleation of the circular muscle layer was performed by electrocautery in this blue marked plane, without accidental opening of the mucosa. Fourth, the opened outer longitudinal muscle was repaired by a running suture using absorbable thread (Figure 3). The resected tumor was put into a surgical bag (Endo Catch™) and removed from the thoracic cavity through an expanded seventh ICS port (when a tumor is too large to be removed through an intercostal space, it is removed through an additional horizontal incision of the upper abdomen through the diaphragm without laparotomy). After carefully investigating for accidental opening of the esophageal mucosa, a single 20-F chest tube was inserted through the ninth ICS port for proper drainage.

## Results

### Surgical outcomes

**Figure 4:**
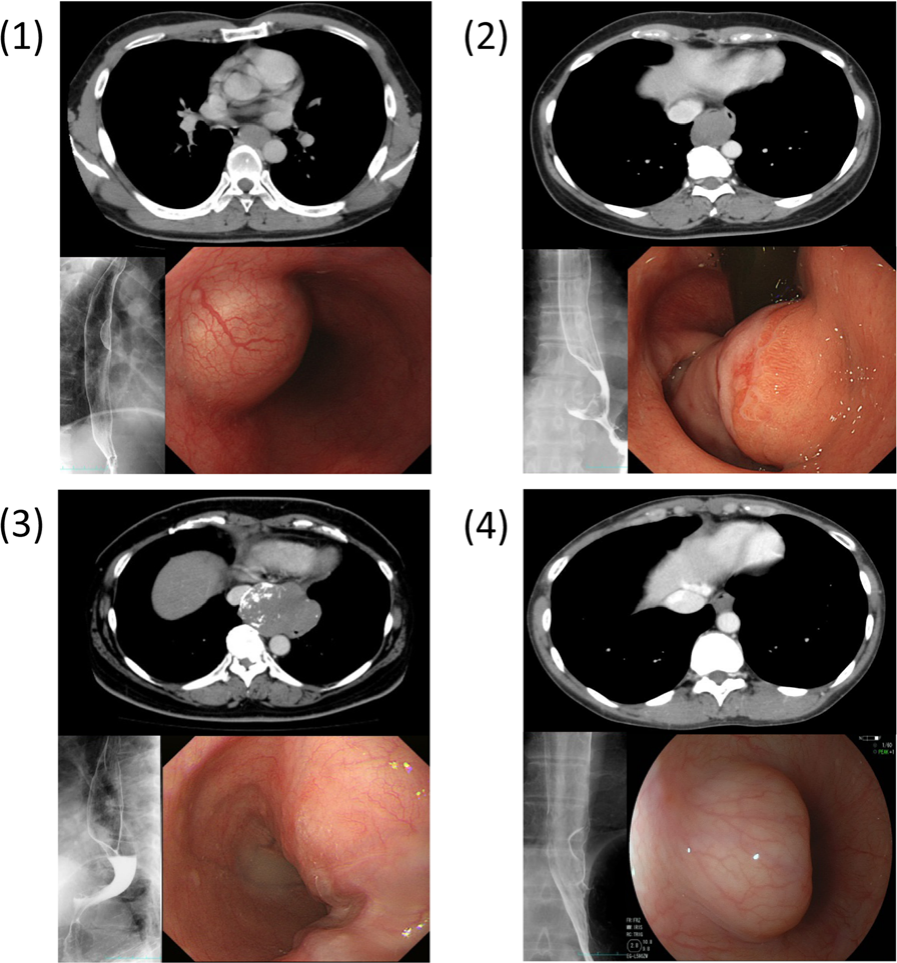
**Findings of contrast-enhanced computed tomography, barium-swallow esophagogram and endoscopy in four cases. (1)** Gastrointestinal stromal tumor located on the left side of the middle third of the thoracic esophagus. **(2)** Tumor located on the lower third of the esophagus was histopathologically diagnosed as leiomyoma. **(3)** Large tumor located on the lower third of the esophagus was histopathologically diagnosed as leiomyoma. **(4)** Tumor located on the lower third of the esophagus was histopathologically diagnosed as leiomyoma.

All four patients underwent complete thoracoscopic enucleation through three ports without access thoracotomy (Figure 4). The tumors of patients 1 and 2 were resected from the port sites. The huge tumor of patient 3 was resected without laparotomy through an additional 4-cm horizontal incision of the upper abdomen just below the costal arch. A summary of clinical characteristics and surgical outcomes of these four patients are shown in Table 1. The mean surgical time was 137.7 min (range, 60–231 min), and the mean blood loss was 21.2 ml (range, 3–65 ml). No intra- or perioperative complications occurred, nor did any surgical deaths.

**Table 1:** **Clinical characteristics and surgical outcomes of four patients**

**Patient**	**Gender**	**Age**	**Location of tumor**	**Size of tumor (cm)**	**Operation time (min)**	**Blood loss (ml)**	**Number of ports**	**Discharge after operation (day)**	**Complication**
1	Male	38	Mt	3.5	127	5	3	10	None
2	Female	43	Lt	5.0	133	12	3	9	None
3	Female	69	Lt/Mt	9.0	231	65	3	6	None
4	Female	38	Lt	3.0	60	3	3	4	None

*Mt* middle third of esophagus, *Lt* lower third of esophagus.

### Pathological diagnosis

One of four tumors was positive for c-kit and CD34 expression, with a mitotic index of 3/50 high-power fields; this tumor was ultimately diagnosed as GIST of the esophagus, with a low risk of aggressive behavior. Three of four tumors were negative for both c-kit and CD34 expression following immunohistochemical investigation. In all cases, resection margins were histopathologically proven as being free from tumors (Table 2).

**Table 2:** **Immunohistopathological staining and pathological diagnosis of the tumor**

**Patient**	**c-kit**	**CD34**	**Desmin**	**S-100**	**SMA**	**Diagnosis**
1	+	+	+	−	+	GIST
2	−	−	+	−	+	Leiomyoma
3	−	−	+	−	+	Leiomyoma
4	−	−	+	−	+	Leiomyoma

*SMA* α-smooth muscle actin.

## Discussion

Because of similar clinical, endoscopic, and radiographic appearances, a correct diagnosis of SMTs originating from the esophagus is often challenging, even with clinically apparent symptoms. Although SMTs of esophageal origin share similar clinical and radiographic characteristics, leiomyoma, leiomyosarcoma, and GISTs are distinct pathologic entities. Therefore, a correct diagnosis of SMTs originating from the esophagus is pivotal for the further management of patients with these entities. To clearly diagnose SMTs originating from the esophagus, several diagnostic methods are available. Because GISTs are fluorodeoxyglucose (FDG)-avid, FDG positron emission tomography (PET) scanning may be used to differentiate GISTs from leiomyoma [8]. Furthermore, GISTs can be reliably identified preoperatively by endoscopic ultrasound (EUS)-guided FNA. However, even EUS-guided FNA lacks the capacity to differentiate between benign and malignant tumors, and normal findings do not exclude the possibility of malignancy by small specimens [9,10]. Therefore, most common esophageal SMTs are not definitely diagnosed until after resection and subsequent pathological examinations.

SMTs larger than 3 cm in diameter suggest the need for treatment due to their high risk of malignancy [1]. Thus, we suggest that if SMT meets the following criteria, surgical resection should be recommended for diagnosis and treatment: (1) the tumor causes symptoms, (2) tumor size is larger than 3 cm, (3) radiologic examination reveals a malignant finding or suspected malignancy, and (4) cytological or pathological examination reveals a malignant finding or suspected malignancy.

Recent advances in thoracoscopic surgery enable us to minimize the procedure to remove SMTs originating from the esophagus. However, enucleation of thoracic esophageal SMTs through a thoracoscopic approach occasionally results in accidental opening of the esophageal mucosa [5,6]. Minimally invasive esophageal surgery such as thoracoscopic esophagectomy with patients in a prone position has demonstrated sufficient short-term outcomes and oncological results [7]. This surgical approach has gained widespread acceptance not only for thoracic esophagectomy but also for enucleation of SMT originating from the thoracic esophagus. Yamada et al. reported thoracoscopic enucleation of esophageal GIST using the prone position with excellent postoperative outcomes [11]. Jeon et al. also demonstrated the safety and efficacy of video-assisted thoracic surgery for enucleation of esophageal submucosal tumors [12]. Furthermore, we combined thoracoscopy with esophagoscopy for maximized complete resection of the tumor, while minimizing exposure of the esophageal lumen by making blue-colored resection layer in the submucosa using endoscopy. Moreover, using our hybrid surgery, thoracoscopic enucleation can be easily performed through only three access ports, which has the advantage of reducing postoperative pain.

## Conclusions

This approach is less invasive and safe and facilitates tumor enucleation, resulting in early postoperative recovery. With all these benefits, our hybrid surgical technique has the potential to be highly effective against SMTs originating from the thoracic esophagus, including GISTs.
